# Perversions with a twist

**DOI:** 10.1038/srep23413

**Published:** 2016-03-30

**Authors:** Pedro E. S. Silva, Joao L. Trigueiros, Ana C. Trindade, Ricardo Simoes, Ricardo G. Dias, Maria Helena Godinho, Fernao Vistulo de Abreu

**Affiliations:** 1 Department of Physics/Institute for Biomedicine -iBiMED, University of Aveiro, Campus Universitario de Santiago, 3810-193, Aveiro Portugal; 2 Institute for Polymers and Composites - IPC/I3N, University of Minho, Campus de Azurem, 4800-058, Guimaraes, Portugal; 3 Departamento de Ciencia dos Materiais and CENIMAT/I3N, Faculdade de Ciencias e Tecnologia, Universidade Nova de Lisboa, 2829-516, Caparica, Portugal; 4 Polytechnic Institute of Cavado and Ave (IPCA), Campus do IPCA, 4750-810, Barcelos, Portugal; 5 Department of Physics/I3N - University of Aveiro, Campus Universitario de Santiago, 3810-193, Aveiro Portugal

## Abstract

Perversions connecting two helices with symmetric handedness are a common occurrence in nature, for example in tendrils. These defects can be found in our day life decorating ribbon gifts or when plants use tendrils to attach to a support. Perversions arise when clamped elastic filaments coil into a helical shape but have to conserve zero overall twist. We investigate whether other types of perversions exist and if they display different properties. Here we show mathematically and experimentally that a continuous range of different perversions can exist and present different geometries. Experimentally, different perversions were generated using micro electrospun fibres. Our experimental results also confirm that these perversions behave differently upon release and adopt different final configurations. These results also demonstrate that it is possible to control on demand the formation and shape of microfilaments, in particular, of electrospun fibres by using ultraviolet light.

The physical mechanisms controlling the shape of elastic filaments are still a matter of intense debate and new findings^1^,^2^. Understanding the elastic behaviour of filamentary materials is very important given the observed diversity in nature^3^,^4^, the mounting interest in the context of nanotechnology^5^,^6^,^7^,^8^ and the need to find simple mechanisms to produce and control the shape of filaments^9^,^10^,^11^,^12^.

In particular, helical structures have drawn considerable attention. First several different mechanisms have been proposed to explain their ubiquitous occurrence in nature. They could be formed by entropic forces^13^, due to intrinsic torsion^14^,^15^ or buckling induced instabilities^16^,^17^. Helical structures can also appear in different arrangements. For instance, proteins are well known for having complex structures, with helices intertwined with beta sheets or other less regular but equally well defined structures. Their organization is crucial for establishing protein function^18^. In polymer science, it is always a challenge to tune the shape of filaments. This can be accomplished by the action of diverse external stimuli such as temperature, polarity or metal ions^19^.

Helical structures have been also obtained with a variety of techniques in nanotechnology. Helices in carbon nano-materials were first observed and reported as “an unusual occurrence” by Davis *et al.*^20^ in 1953. Since then, many strategies have been devised to produce materials with different coiling. Chemical vapour deposition allows the production of high quantities of helices. The strategy consists in using an unequal extrusion of carbon from a catalyst surface^21^,^22^,^23^. This technique allows producing helical structures with different shapes simply by changing the catalyst grain morphology. Helices can also be grown by considering that catalyst particles are affected by van der Waals forces between the fibre and the surroundings, as in the work of Zhang *et al.*^24^. It has been advocated that these structures can find a wide range of applications, such as nanosprings^25^,^26^, electronic circuit components^27^,^28^ and in reinforcement composites^29^.

Considerable work has also been done to obtain helical filaments by electrospinning. However, in many cases the helical structures become flattened as they land on the target^30^,^31^,^32^. This is not the case reported by Chen *et al.*^33^, who produced coiled core-shell fibres by coaxial electrospinning. A simpler technique has been proposed by Trindade *et al.*^34^, using ultraviolet (UV) irradiation upon the fibres. In both techniques it is the differential shrinkage of the elastomeric polymer that induces coiling due to buckling. In this work we will use a procedure adapted from the latter technique.

An interesting feature that can be observed in all these helical structures is the inversion of the twisting direction, known as a perversion. Perversions appear also in plants when tendrils attach to a support and shorten to direct the plant towards the attachment point. Perversions arise when filaments curl having both ends clamped. In simple terms, perversions can be seen as structures connecting helices with opposite handedness.

Considerable work has been devoted to better understand perversions and its relevance. On the mathematical side, Goriely and Tabor^16^ discussed the formation of a perversion in the presence of intrinsic curvature using Kirchhoff equations. Later, Domokos and Healey^35^ obtained solutions in the presence of multiple perversions. The occurrence of multiple perversions was also studied both theoretically and experimentally by Huang *et al.*^36^, who showed that a helical filament could display an arbitrary number of perversions depending on the pre-strain imposed on one side of a filament. Perversions arise also when torsion is externally applied on an elastic filament, as discussed by Lazarus *et al.*^2^. Finally, Gerbode *et al.*^1^ used a simple experimental set-up to demonstrate the ubiquitously presence of perversions in tendril coiling. In all these works, perversions are described as having the same geometries and impinging the same elastic properties to the elastic filaments. Next we will show that this view can be considerably enlarged.

## Motivation

**Figure 1:**
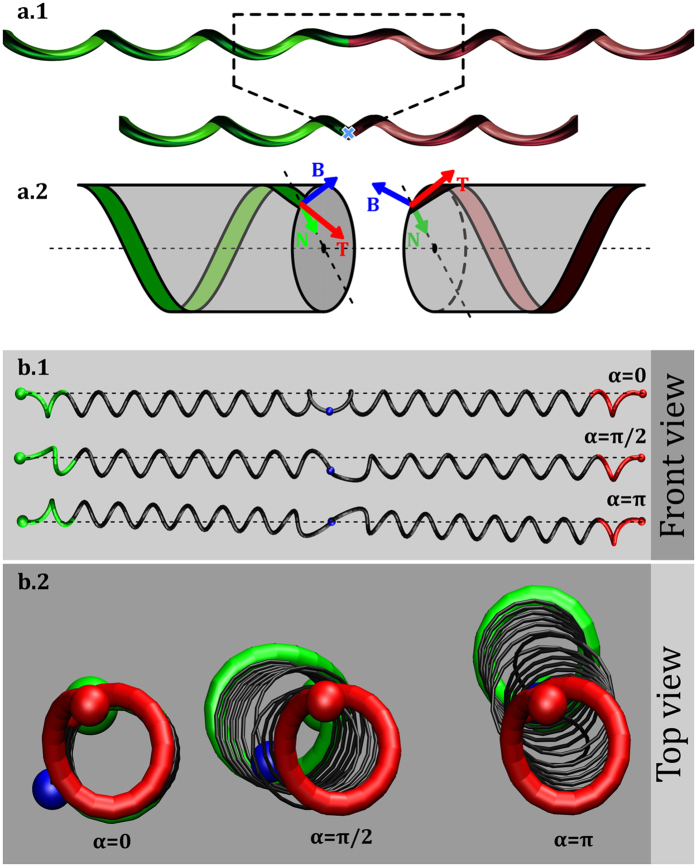
Generalized perversions in helical filaments. **(a.1)** Illustration of the simplification introduced in the model: the initial filament is reduced to three elements, two perfect helices with opposite handedness (right and left handed depicted in green and red respectively) and a perversion point (blue cross). **(a.2)** FS frames at the points connecting the perversion for a symmetric perversion, *α* = 0. Front **(b.1)** and top **(b.2)** views of 3 types of perversions, with *α* = 0, *π*/2 and *π*. In the front view, it is clear that symmetric perversions display an *M* shape and the filament is symmetric relatively to the axial plane passing through the perversion centre. By contrast, antisymmetric perversions, *α* = *π*, display an *N* shape and are antisymmetric instead. The top view clearly shows that symmetric perversions keep the two helices axis remain steady while for antisymmetric perversions helices axis tilt relatively to the direction of release. For other perversions (for instance, with *α* = *π*/2), a combination of the two effects occurs.

In this work we questioned whether tendril perversions are the only structures linking helices with opposite handedness. A simple theoretical argument suggests that other possibilities exist. Consider the example in Fig. 1, where two helices with opposite handedness are linked by a perversion at the centre and the ends are held so that they do not rotate. Our analysis consists in reducing the complexity of the system to a set of two helices with opposite handedness linked at a single point. This linking point represents a region of the curve equidistant relatively to the centre of the perversion, perturbed by the presence of the perversion. Thus, outside this region the curve behaves approximately as two perfect helices. A simple way of describing helical curves uses the Frenet-Serret (FS) frame, *Q* = [**T**, **N**, **B**], where **T** is the unit vector tangent to the curve, **N** is the normal vector and **B** is the binormal vector given by the cross product of **T** and **N**. The evolution of the FS frame along the curve can be written in terms of the Darboux vector, **Ω**, through the continuous differential equations 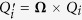. Left and right handed helices have constant curvature, *κ*, and torsion, *τ*. They differ only on the Darboux vector, through a sign change in the torsion factor: **Ω** = *κ***B** ± *τ***T**.

In our simplified model, these equations must be discontinuous at the linking point, and be capable of relating Darboux vectors and FS frames of the two helices, as illustrated in the example in Fig. 1a.2. Since in any helix, normal vectors always point towards the centreline, the normal vector is perpendicular to the twisting vector which is always directed along the helix axis. In this particular case, the left helix tangent vector can be obtained from the right helix tangent vector by a rotation around the normal by 2*θ*, where *θ* is the angle between the tangent and the twisting vector. As a result, in the simplified model, the FS equations can be rewritten as:


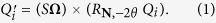


The only difference between this equation and the conventional FS equations is the application of the two transformations, *R*_**N**,−2*θ*_ and *S*, at the perversion point. *R*_**N**,−2*θ*_ denotes a rotation of the FS frame around the normal and *S* changes the handedness of the helix, *S***Ω** = *S*(*κ***B** + *τ***T**) = *κ***B** − *τ***T**.

The important observation made in this work is that any further rotation of the FS frame around **Ω** is still compatible with existence of a right helix with the opposite handedness. That is, we can generalize the previous equation to:


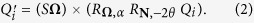


Thus, *α* = 0 corresponds to the description of perversions as are usually described. However, this equation suggests that other types of perversions can also exist. After the second rotation operating on the FS frame, the right helix conserves the same direction of the rotation axis but its position can change. On Fig. 1b we illustrate filaments with different types of perversions. Different perversions induce different symmetry properties and different shapes (Fig. 1b). For *α* = 0 and *α* = *π*, filaments are respectively symmetric and antisymmetric relatively to the axial plane passing through the perversion centre. For this reason, we named perversions with *α* = 0 and *α* = *π* as symmetric and antisymmetric perversions.

Importantly, these symmetry properties are maintained also during force-extension experiments, as long as helices are aligned along the extension axis. As a result, symmetric perversions will keep both helices axis aligned and the perversion rotates around the axis while the number of loops changes. By contrast, for antisymmetric perversions, the helices axis tilt relatively to the direction of release (see Fig. 1b.2 and supplementary materials SM1). When the number of loops in the helices changes during elongation, the perversion rotates around itself while its location remains static for antisymmetry to be maintained. More complex dynamics and configurations take place for perversions with other values of *α*. Nevertheless, in general terms, for *α* ≠ 0 helices axis are tilted and their axis locations are related by *α*. This can be understood taking into account that the total twist does not change during force-extension experiments since a moment is applied at both clamped ends^37^. Given that the twist developed by the two helices is symmetric, they cancel each other, and consequently the total twist of the filament is equal to *α*, the total twist of the filament. This implies that FS frames at the ends of the filament are rotated by *α* around **Ω**, which establishes the different orientation of the helices.

The challenge reported in this paper has been to reproduce these generalized type of perversions. We focused on generating perversions with *α* = 0 and *α* = *π*, the two most distinctive types of perversions. Symmetric perversions appear naturally when filaments held on both ends twist to adopt a helical shape, as reported in^17^,^37^. To generate antisymmetric perversions we noted, that after the *α* = *π* rotation, the normal vector switches direction. This implies that the bending of a filament at this point must change abruptly. This consideration offered a perspective on how to make materials with these properties: by intrinsically changing the curvature of filaments in specific regions. In the next section we describe how microfilaments with the two types of perversions were created. Afterwards we will further discuss the physical behaviour of these filaments.

## Results

### Writing the intrinsic curvature on microfilaments

**Figure 2:**
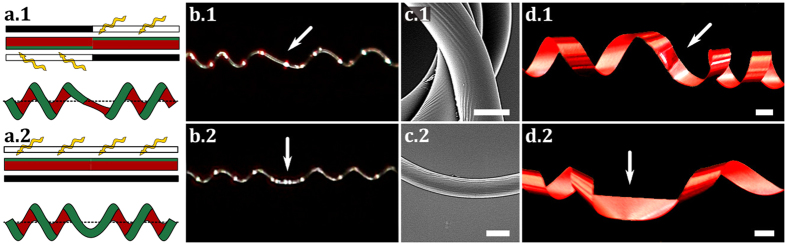
Generation of antisymmetric and symmetric perversions. (**a**) Sketches illustrating UV writing on fibres, for generation of **(a.1)** antisymmetric and **(a.2)** symmetric perversions. Elastic electrospun filaments after UV light exposition and fibre relaxation for **(b.1)** antisymmetric and **(b.2)** symmetric perversions. White arrows point to the perversion centres. (**c**) SEM pictures for the two perversion types. **(c.1)** In antisymmetric perversions wrinkles induced by UV light on the outer surface of the fibre changes side while **(c.2)** in symmetric perversions they remain on the same side. (**d**) The two types of perversions can be observed in gift-wrap ribbons. The antisymmetric perversion is obtained by glueing the opposite sides of two ribbon halves. Scale bar represent 5 *μ*m in (**c**) and 1 cm in (**d**).

Elastic microfilaments, with intrinsic curvature, were obtained from electrospun fibres produced from two pre-polymers with different functionalities (see [materials]Materials), which generate a crosslinking network after formation of the fibres. Two flat parallel electrodes were used to collect stretched fibres. If fibres are released at this point, no coiling is observed, but only bending due to gravity (see supplementary video SV9). Application of UV light established an asymmetry between the two sides of a filament by further crosslinking of the outer surface of the fibres double bonds, existing along one of the repetitive units^38^. In Fig. 2a, sketches represent stretched fibres irradiated on opposite alternate sides (a.1) or on the same side (a.2) along the main axis of the filament. The higher crosslinking density at the top layer of the fibre relatively to the remaining soft material creates a differential shrinkage. This is responsible for the intrinsic curvature of the filament. This way we can write the outer surfaces of the stretched filaments with UV. Upon relaxation of the fibres clamped at both ends, coiling of filaments creates helical structures separated by perversions which can exhibit different symmetries depending on the UV writing. In Fig. 2b.1 a spontaneous antisymmetric perversion appears in an elastic filament, treated as described in (a.1), after being released. The *N* shape of this antisymmetric perversion is clearly different from *M* of the (conventional) symmetric perversion in Fig. 2b.2, for a filament irradiated only on one side (a.2).

The UV writing wrinkles the fibres surfaces as can be observed in Scanning Electron Microscopy (SEM) images (see Fig. 2c). In filaments with antisymmetric perversions wrinkles switch side at the middle of the perversion, (c.1), while in filaments with symmetric perversions wrinkles are always on the same side (c.2). The same kind of antisymmetric perversions can be observed in gift ribbons (Fig. 2d). After sweeping the dull side of the stripe with a scissors blade the ribbon acquires intrinsic curvature and forms a helix. Stretching while removing the loops of the strip, will lead to a straight configuration. The release of the ribbon with both ends clamped creates perversions with a visible switch of the helical handedness^39^. If the fibre is cut in two halves and glued together switching the two sides, an antisymmetric perversion appears as shown in Fig. 2d.1. For a matter of comparison, a ribbon with a symmetric perversion is shown in Fig. 2d.2.

### Release of filaments with intrinsic curvature

**Figure 3:**
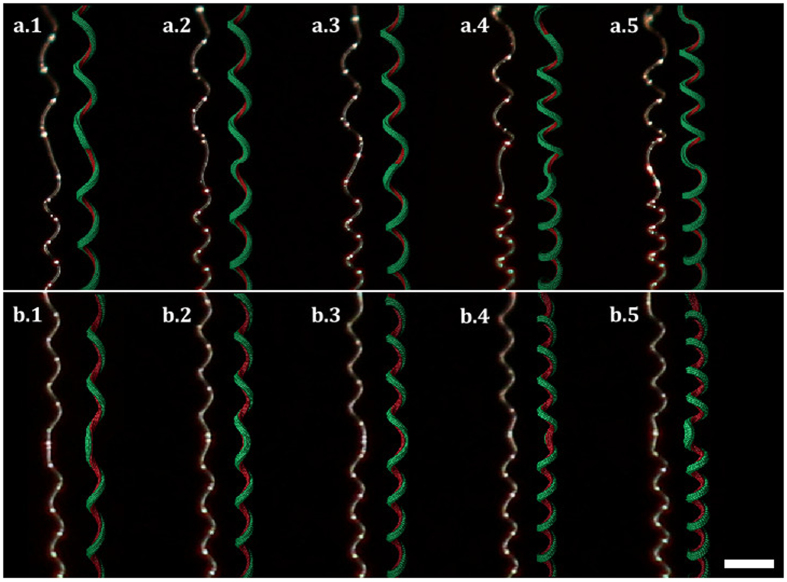
Evolution of the shape of elastic filaments with intrinsic curvature upon release. Photos taken from experimental fibres (left filaments) and from molecular dynamics simulations (right filaments), displaying how (**a**) antisymmetric and (**b**) symmetric perversions curl during release. Simulations describe qualitatively well the experimental behaviour of both fibres.

Antisymmetric and symmetric perversions were ‘printed’ in the elastic stretched filaments as described in the methods section. Fibres were released at a controlled rate, with both ends constrained from rotating. In Fig. 3, results obtained with a filament irradiated for 24 h, initial length *L* = 1 cm and diameter *D* = 7 *μ*m, are shown as well as results obtained with numerical simulations with physically plausible parameters. As shown in Fig. 3, simulations reproduce well the coiling of the fibre in the presence of the perversion when the load decreases (see videos SV2 and SV5 in supplementary materials).

**Figure 4:**
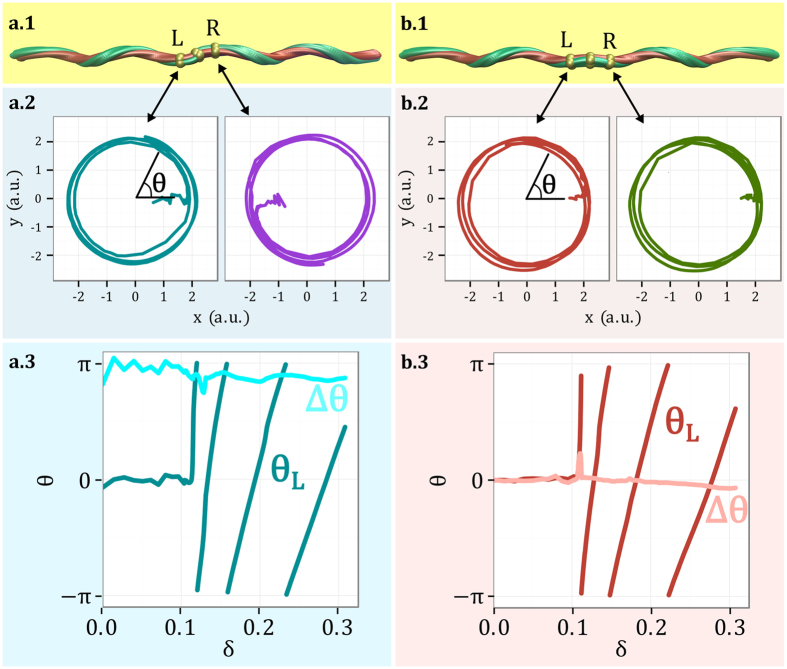
Detailed analysis of the movement of two symmetric points close to the perversions centres during unloading. (**a**) In antisymmetric perversions, the two points, designated by *L* (Left) and *R* (Right), move in opposite directions while (**b**) they move in the same direction in the symmetric perversion. Here *θ* represents the angular position in polar coordinates. These numerical results were obtained during fibre release, here parametrised by 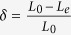, where *L*_0_ represents the initial fibre elongation and *L*_*e*_ the end-to-end distance. From (**a.3,b.3**) it is clear that upon release the phase difference, Δ*θ*, between the two points is approximately constant and equal to *π* or 0 for the antisymmetric or symmetric perversions, respectively.

The movement of the filament during unloading must be compatible with the symmetry of the perversion. Antisymmetric links force right and left helices to rotate in antiphase around the line connecting the two ends (Fig. 3a). By contrast, the symmetric perversion promotes the rotation in phase of the two helices (Fig. 3b). To further enlighten the difference in the movements in the presence of the two types of perversions, numerical simulations were performed leading to the detailed analysis in Fig. 4. In this figure, the movement of two points, symmetrically disposed relatively to the perversion centre, is depicted and clearly shows that the two points move around the axis, defined by the two ends, either symmetrically or antisymmetrically, in accordance with the perversion name. An interesting consequence of the antisymmetry property of the antisymmetric perversion, is that the point at the centre does not change position. This is also in agreement with our experimental observations and with the simplified model. Another interesting result, which is also in agreement with our experimental observations, is that the curling speed decreases with the unloading.

**Figure 5:**
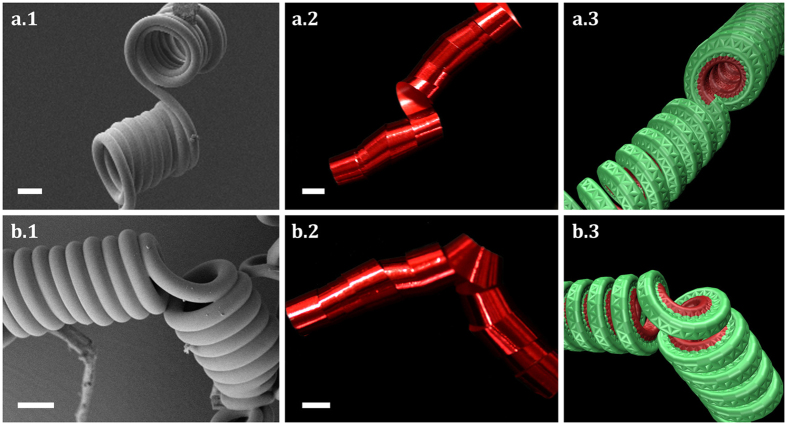
Final configuration for helices connected by antisymmetric and symmetric perversions. After complete relaxation of the elastic filament, the filaments adopt distinctive shapes. For antisymmetric perversions (top figures) helices remain aligned while they bend for symmetric perversions (bottom figures). Scale bar represent 10 *μ*m in (**a**) and 1 cm in (**b**).

The dynamics observed in fibres with perversions is not the only distinctive feature. Equilibrium configurations are also conspicuously different. In Fig. 5a, SEM pictures of unstrechted microfilaments written with the two types of perversions are shown. Symmetric and antisymmetric perversions are clearly different, a result that can also be readily observed either with ribbon strips or in numerical simulations. This shows that the difference between the two types of perversions is a robust result observable at different scales. In antisymmetric perversions helices remain aligned as a result of the fact that there is no bending at the antisymmetric perversion centre. By contrast, symmetric perversions tend to bend the link between the two helices, creating an angle between them.

Finally, as antisymmetric perversions require a switch of the intrinsic bending properties, the perversion can only occur at specific locations. This also means that they should not be expected to be as ubiquitous in nature, but, on the other side, they can be engineered on demand. This contrasts with symmetric perversions which can travel along the material during their formation and hence have a more delocalised nature.

## Materials and Methods

### Materials

The elastic filaments were obtained from electrospinning by using a methodology similar to the one described previously in literature^34^. The elastic behaviour of the filaments was controlled by the relative amount of the pre-polymers used for preparing the starting solution. The first network was prepared from the reaction of poly(propylene oxide)-based triisocyanate-terminated prepolymer (PU) and hydroxyl-terminated polybutadiene (PBDO), the second crosslinking reaction on the top of the filaments was promoted by UV irradiation, according to the procedure and details described previously, for 40% w/w of PU and 60% w/w of PBDO^34^. The completion of the first reaction was achieved after the formation of the PU/PBDO electrospun fibres. The precursor solution with the two prepolymers, dissolved in toluene, was poured into a 1 ml syringe fitted with a 25-gauge needle and an infusion syringe pump (model KDS100) was used to control the solution feed rate (0.8 ml h^−1^). A conducting ring of 15 cm diameter was held coaxially with the needle tip at its centre, and electrically connected to it and to the positive output of a high-voltage power supply (Glassman EL 30 kV). The solution was accelerated towards a suspended collector target consisting of two parallel, flat metallic bars, by the action of an electric field applied between the syringe tip and the rotating target. A sample holder, made using acetate sheets cut with a precision cutting machine (Silhouette Portrait) was placed between the metallic bars. Optimal electrospinning conditions for this system were found to be: voltage at 14 kV for a syringe tip-target separation of 15 cm, relative humidity 70%. The filaments were UV irradiated (*λ* = 254 nm, Intensity = 10^−5^ mW/cm^2^, lamp to sample distance *L*_*UV*_ = 20 cm) during 24 h and extracted for 24 h (T = 50 °C) (Soxhlet extractor) in toluene, followed by drying in an oven for 48 h at 50°, before being observed by SEM and POM (Polarized Light Microscopy). To produce symmetric perversions, fibres were irradiated with UV light only on one side of the surface. Antisymmetric perversions were produced irradiating the two sides of the fibre but on complementary regions using two irradiation cycles. In the first cycle, a mask made with an opaque card, cut with a precision cutting machine, protected sections of the fibre from UV light. For the second irradiation cycle the sample was flipped and the complementary mask was used to apply UV light to the other side (see Fig. 2a and illustration in supplementary materials SM2)

### Unloading procedure and characterization of the microfilaments

The experimental observations of the unstretching of the filaments were made under axial loading. This was accomplished by using an extensometer from Rheometric Scientific (Minimat Firmware Version 3.1) coupled to a Canon EOS550D Camera equipped with a macro lens (EFS 60 mm). The lengths and diameters of individual filaments visible in these SEM images were measured with ImageJ software (version 1.48, http://imagej.nih.gov/ij/) and scaled according to the magnification quoted by the microscope software. Optical observations were achieved by using a reflection and transmission mode Olympus BX51 microscope equipped with an Olympus DP73 camera. SEM was used to image the morphology features of the elastic filaments with an Auriga crossbeam (SEM-FIB) workstation instrument (Carl Zeiss) equipped with an Oxford energy dispersive X-Ray spectrometer. The SEM images under the in-lens mode have been carried out with an acceleration voltage of 2 kV and aperture size of 30 *μ*m. The substrates with filaments were glued on to aluminium stubs using a double-sided carbon tape and coated with a thin carbon layer (<20 nm) using a Q300T D Quorum sputter coater.

### MD simulation

To study the dynamics of elastic filaments we developed molecular dynamics simulations using LAMMPS (Large-scale Atomic/Molecular Massively Parallel Simulator, http://lammps.sandia.gov) platform^40^,^41^,^42^. Elastic fibres were modelled by arranging beads in hexagonal close packed lattices (see SFig. 11 in supplementary materials SM3) bonded by harmonic potentials, *V*_*i*_ = *k*_*h*_/2(*l* − *l*_*i*_)^2^, where *k*_*h*_ is the elastic constant and *l*_*i*_ the equilibrium bond distances. Filaments had initial length *L* = 1200 *σ* (LJ units, see http://lammps.sandia.gov/doc/units.html) and 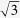 by 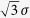 of cross-section. Intrinsic curvature was produced by pre-straining one side of the rod. This was accomplished by changing the equilibrium bond distances as shown in supplementary materials SM3. Different helical curvatures were obtained by controlling the pre-strain amplitude. The intrinsic curvature was selected using the relation 
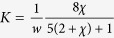
 (as discussed in Supplementary Materials SM3), where *w* is the fibre cross-section width and *χ* is the initial pre-strain. Simulations were deterministic and did not include temperature effects. The integration of the equations of motion used a NVE integrator (Verlet/Leap-frog method) to update beads positions and velocities on each timestep (stepsize of 1 × 10^−3^ *τ*). Helical structures were formed by releasing the filament at constant rate and with the ends set to not rotate. Different helical curvatures were established by controlling the pre-strain amplitude. To generate antisymmetric perversions the pre-strained side of the filament was changed at specific regions.

## Conclusions

In this work we have proposed that a whole range of perversion geometries can exist in elastic filaments. We have given special attention to the demonstration that two extreme types of perversions, denominated symmetric and antisymmetric, exist in practice, and in particular at the microscale. These two perversion types exhibit distinct properties. Symmetric and antisymmetric perversions have different shapes (*M* or *N*), upon release they rotate around the centreline in or out of phase relatively to their centres and adopt different final configurations (bent or aligned). These properties were put in evidence through numerical and experimental work and are summarized in the supplementary video SV1.

Both types of perversions were reproduced with PU/PBDO electrospun filaments using irradiation with UV light. Irradiation with UV light was crucial to create an asymmetric contraction of the fibre relaxation through a higher crosslinking of the elastomer network. Irradiation of UV light on selected areas worked as a printing tool allowing to create antisymmetric perversions on demand. Our methodology can be applied to other type of elastic fibres only by playing selectively on the crosslink density across the filament. In this way, threads can be self-shaped to spontaneously produce symmetric or antisymmetric perversions linking right and left handed helices. Since linked helices adopt different configurations for the different perversions, perversions can be used to assemble fibres in different types of structures, which could find applications in nanotechnology or in the textile industry.

The study presented here focused on describing the new symmetry properties of these generalized type of perversions. Further studies are still necessary to describe their shape in detail, to enlighten how the different type of perversions appear, how they can coexist and interact, to analyse how stresses are distributed in fibres and how perversions may lend materials with new properties. Some of these issues will be pursued in future studies.

## Additional Information

**How to cite this article**: Silva, P. E. S. *et al.* Perversions with a twist. *Sci. Rep.* **6**, 23413; doi: 10.1038/srep23413 (2016).

## Supplementary Material

Supplementary Information

Supplementary Video 1

Supplementary Video 2

Supplementary Video 3

Supplementary Video 4

Supplementary Video 5

Supplementary Video 6

Supplementary Video 7

Supplementary Video 8

Supplementary Video 9
