# Supplementary material for: Perversions with a twist
Source: Sci Rep. 2016 Mar 30;6:23413. doi: 10.1038/srep23413 (PMC4812244; doi:10.1038/srep23413)
Supplement: Supplementary Information [file srep23413-s1.pdf]

Perversions with a twist

Supplementary Materials

Pedro E. S. Silva[1], Joao L. Trigueiros[2], Ana C. Trindade[3], Ricardo Simoes[2,4], Ricardo G. Dias[5], Maria Helena Godinho[3], and Fernao Vistulo de Abreu[1,*]

[a]Department of Physics/Institute for Biomedicine - University of Aveiro, Campus Universitario de Santiago, 3810-193, Aveiro Portugal.
[b]Institute for Polymers and Composites - IPC/I3N, University of Minho, Campus de Azurem, 4800-058, Guimaraes, Portugal.
[c]Departamento de Ciencia dos Materiais and CENIMAT/I3N, Faculdade de Ciencias e Tecnologia, Universidade Nova de Lisboa, 2829-516, Caparica, Portugal.
[d]Polytechnic Institute of Cavado and Ave, Campus do IPCA, 4750-810, Barcelos, Portugal.
[e]Department of Physics/I3N - University of Aveiro, Campus Universitario de Santiago, 3810-193, Aveiro Portugal.
[*]fva@ua.pt

SM1: Videos Overview and Legends

For a clearer understanding of the phenomena reported in this work we display a set of figures from accompanying videos and respective video legends.

Video **SV1**: Comparative overview of symmetric and antisymmetric perversions obtained in computer simulations, experiments with electrospun fibres and gift ribbons.

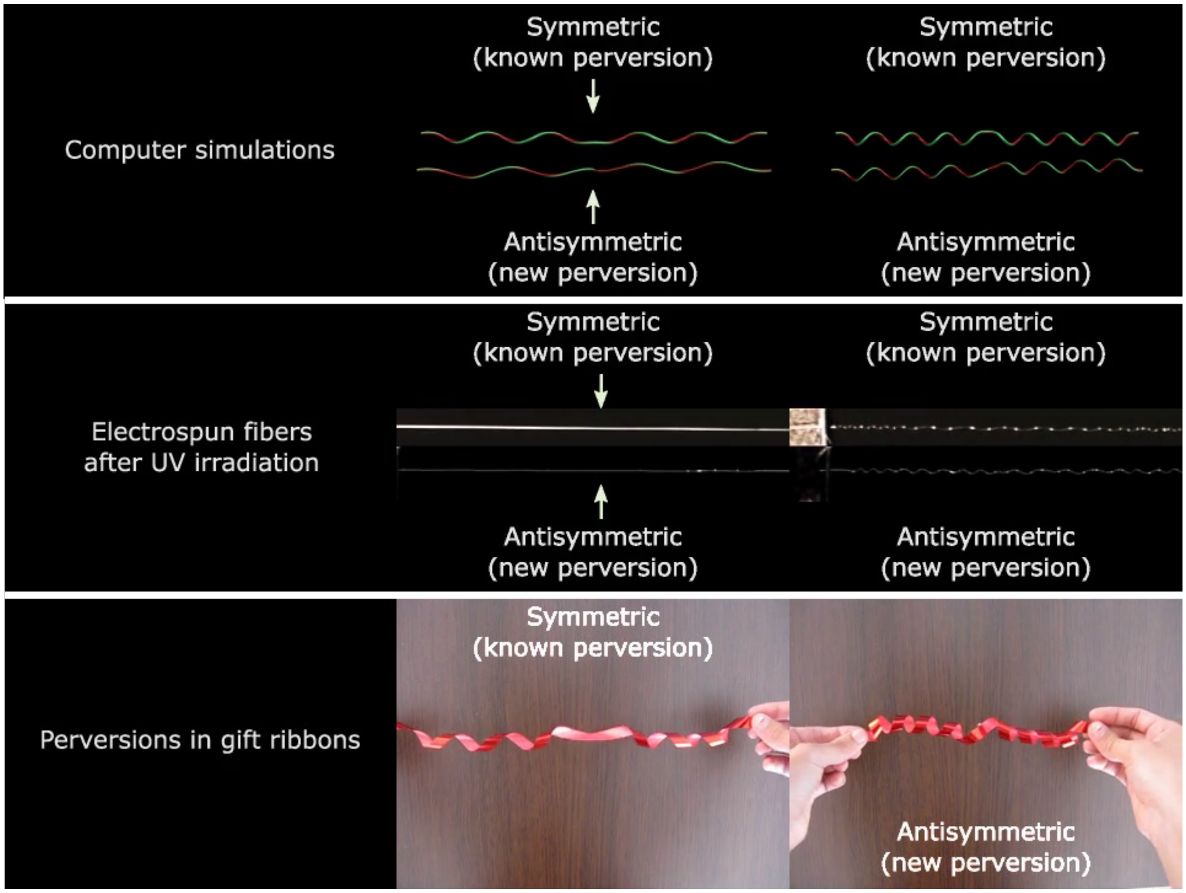

SFig. 1: Snapshots from the video overviewing symmetric and antisymmetric perversions.

Video **SV2**: Simulations obtained releasing a pre-strained fibre with both ends fixed.

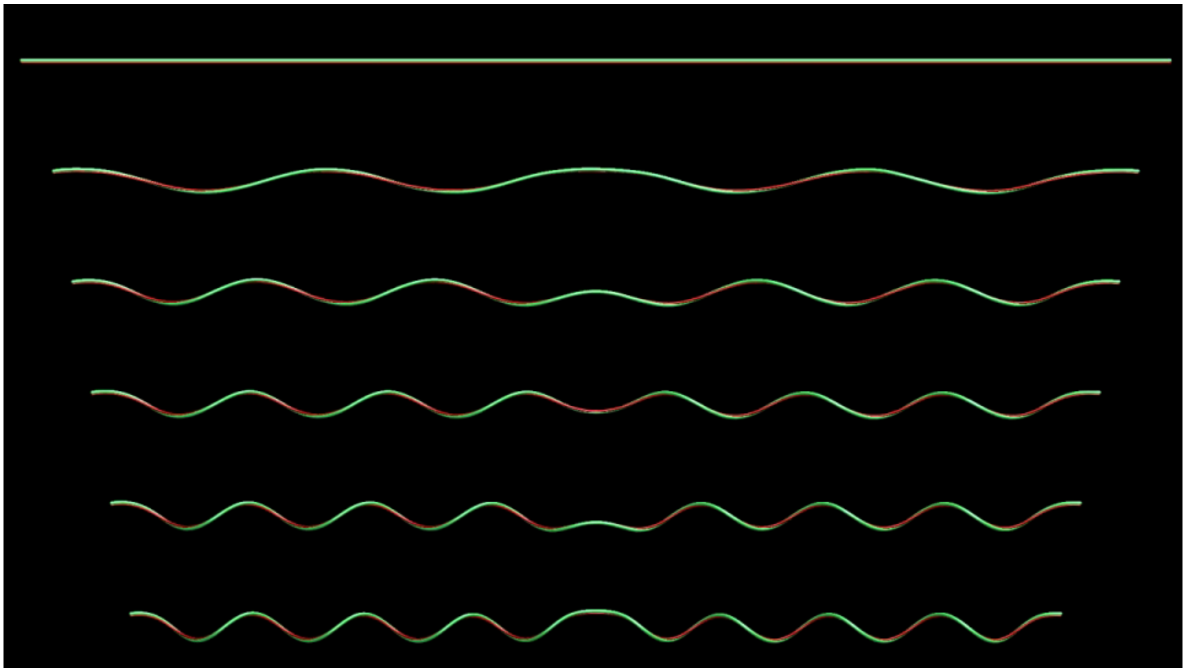

SFig. 2: Sequence of images from supplementary video **SV2**. A symmetric perversion appears in the centre of the filament. Pre-straining is depicted in red.

Video **SV3**: Top view of the previous simulation **SV2**.

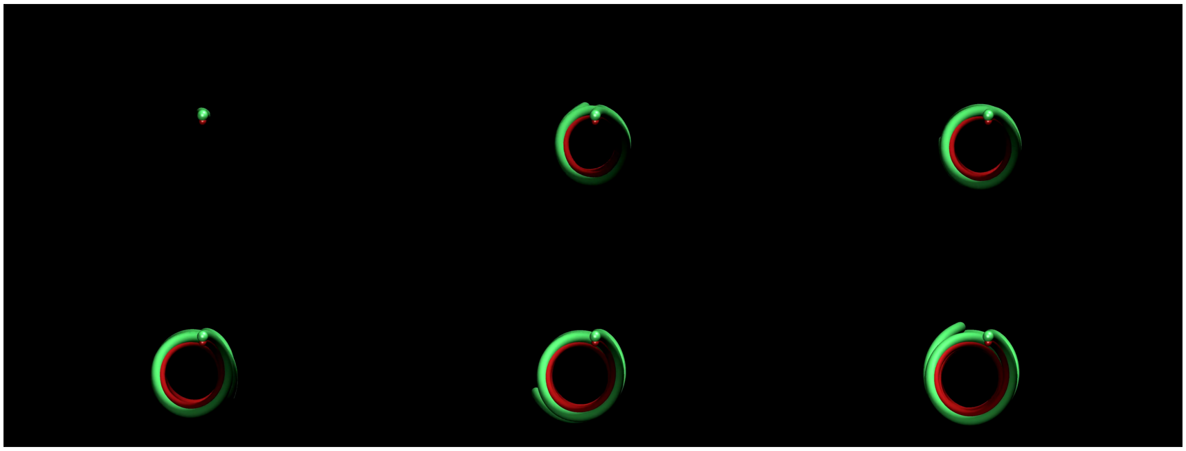

SFig. 3: The top view shows the symmetric perversion revolving around the centreline of the filament (supplementary video **SV3**).

Video **SV4**: Another view of simulation **SV2**.

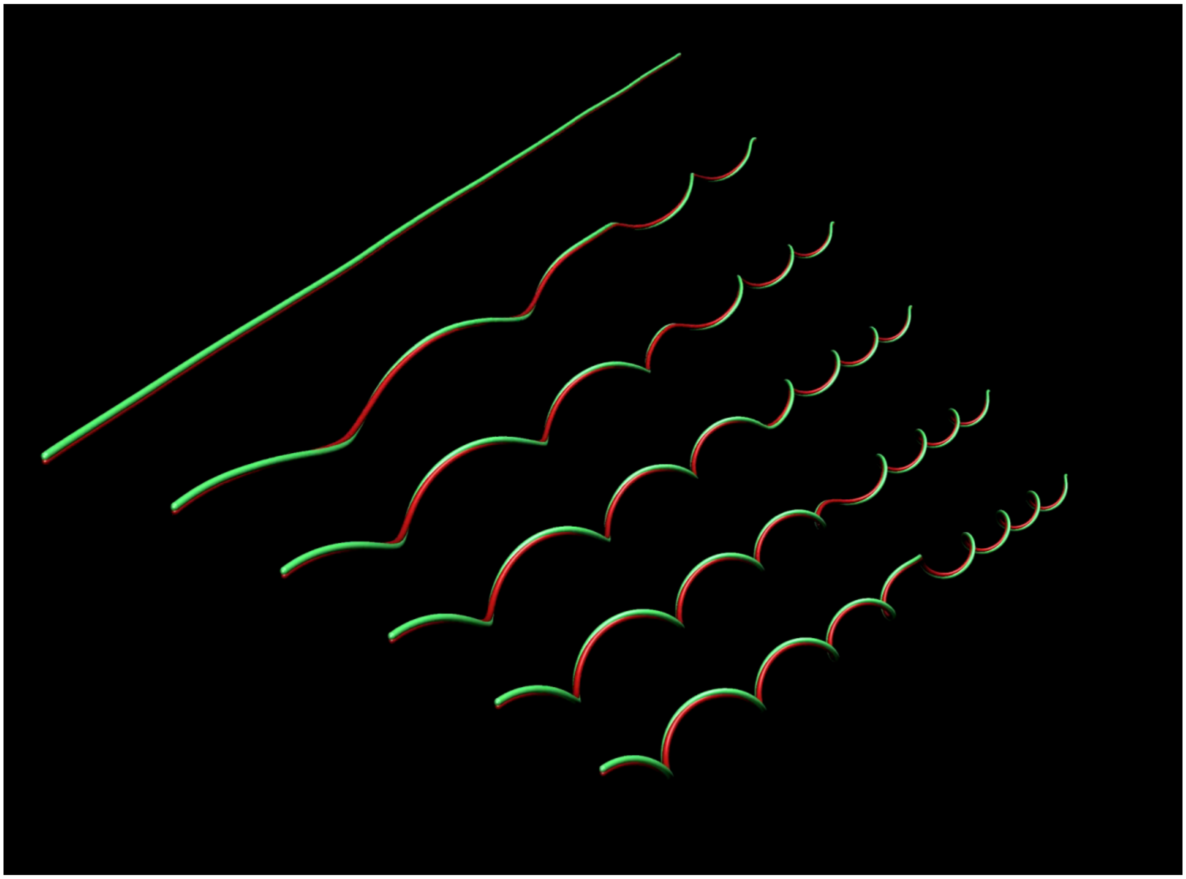

SFig. 4: View from another angle of the same simulation steps illustrated in SFig. **2** (supplementary video **SV4**).

Video **SV5**: Simulations obtained releasing a pre-strained fibre with both ends fixed, but with a change in pre-straining in the middle of the fibre.

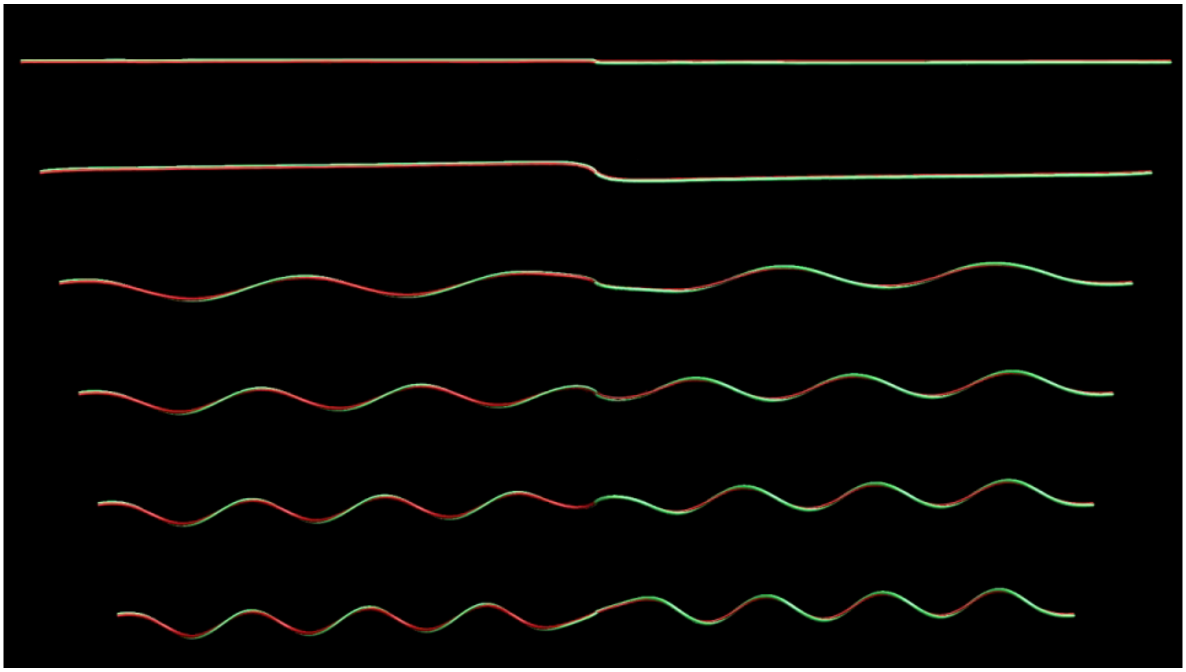

SFig. 5: Sequence of images from supplementary video **SV5**. An antisymmetric perversion arises at the centre of the filament. In contrast to the symmetric case, the centre of the antisymmetric perversion remains aligned with the filament ends.

Video **SV6**: Top view of the previous simulation **SV5**.

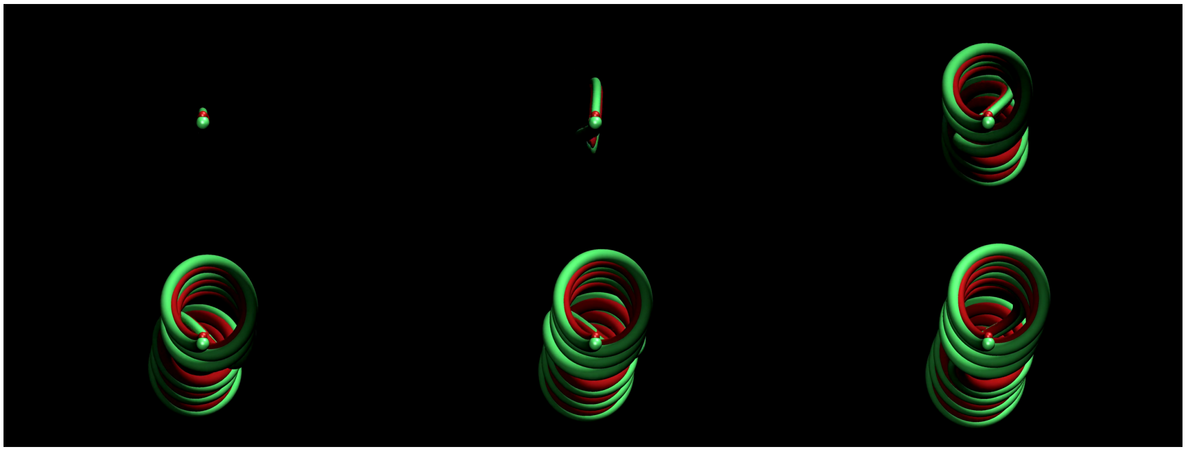

SFig. 6: In antisymmetric perversions, helices centrelines on the two sides of the perversion are uncentred (supplementary video **SV6**).

Video **SV7**: Another view of simulation **SV5**.

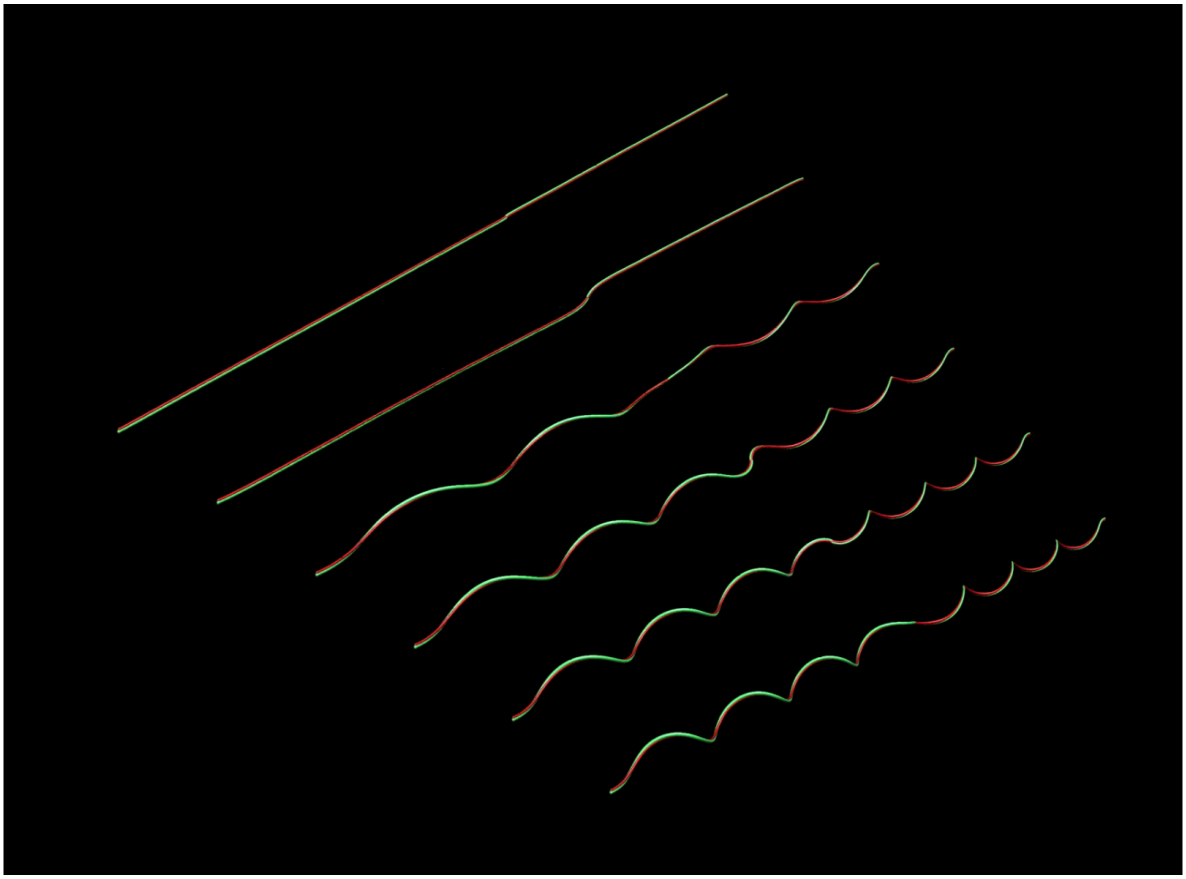

SFig. 7: View from another angle of the same simulation steps illustrated in SFig. **5** (supplementary video **SV7**).

Video **SV8**: View along helices centreline of simulation **SV5**.

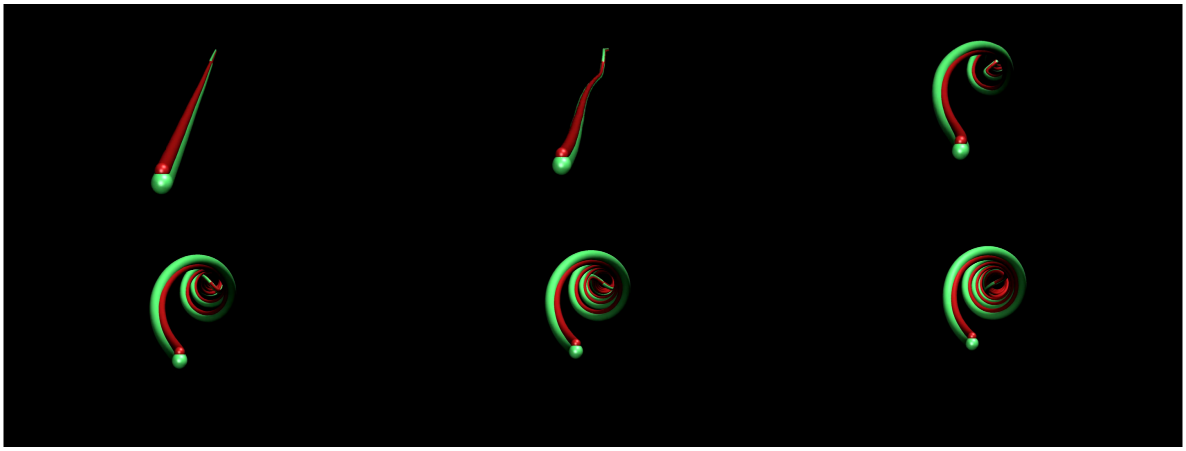

SFig. 8: View along helices centreline of the same simulation steps illustrated in SFig. **5** (supplementary video **SV8**).

Video **SV9**: Release and extension of a electrospun fibre with no UV irradiation.

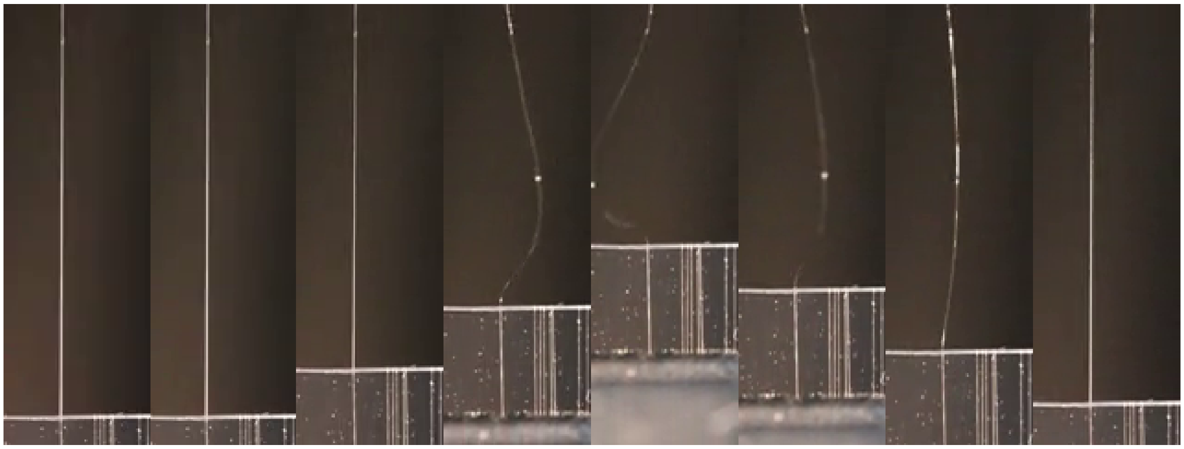

SFig. 9: Release of a polymeric fibre without UV irradiation. The fibre bends due to gravity but shows no sign of intrinsic curvature.

SM2: Experimental procedure

In SFig. **10** the several steps involved in sample preparation are depicted.

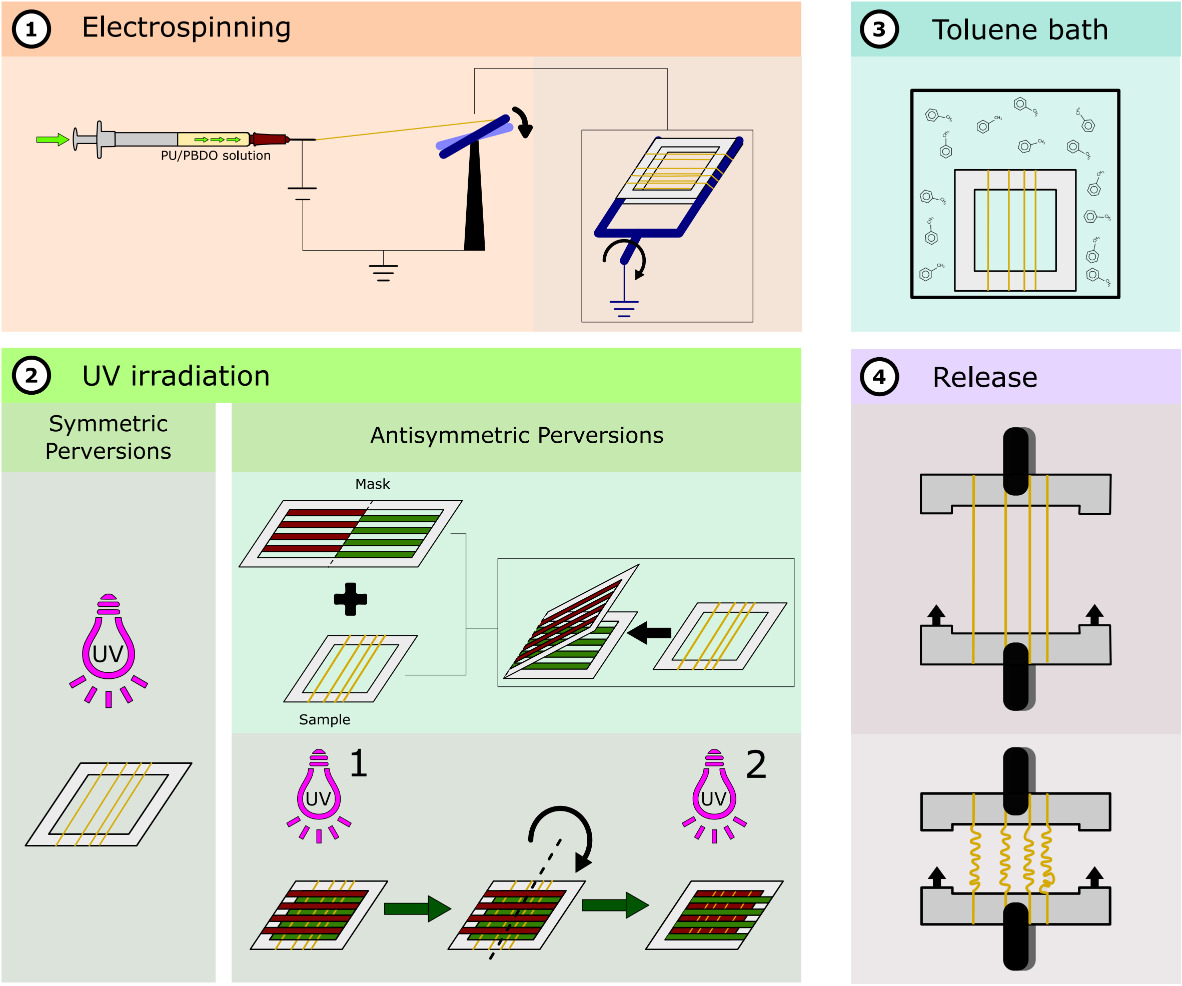

SFig. 10: **Schematic representation of the several experimental steps to produce polymeric fibres with symmetric and antisymmetric perversions.** ① A solution of PU/PBDO dissolved in toluene is accelerated towards a suspended collector target consisting of two parallel metallic bars by action of an electric field applied between the syringe tip and the rotating target. The gel point is achieved after the deposition of the fibres. ② To produce symmetric perversions, fibres are irradiated during 24h on one side with UV light. To produce antisymmetric perversions, two cycles of UV irradiation are applied sequentially on each side; masks protect complementary regions of the fibre from the irradiation. By the action of UV light in the presence of the air oxygen the double bonds of the PBDO were allowed to open and form an additional network on the top of the fibre. ③ UV-irradiated fibres are swollen in toluene for 24h (Soxhlet extractor) to remove the sol fraction. ④ After being dried, fibres are released at a controlled rate, with both ends constrained from rotating.

SM3: Simulations of perversions and relation to experiments

The model described in this paper provides a simple description of the different types of perversions. In order to provide additional evidence for the mechanism proposed to observe the different types of perversions we used LAMMPS (Large-scale Atomic/Molecular Massively Parallel Simulator) to model the dynamics of elastic filaments.

Microfilaments were formed using structures with cross-sections made of $N_w = 3$ by $N_h = 3$ beads along the width and height, respectively (see SFig. **11a**). Beads were connected by two harmonic potentials, denoted a and b, with equilibrium bond distances, l_a and l_b, and illustrated in different colours in SFig. **11**. For sufficiently unmatched bond distances (in our case, with $l_b < l_a$), one side of the filament becomes stretched relatively to the other, and upon release the filament gains a helical shape with an intrinsic curvature K, as well discussed in Liu et al.[?].

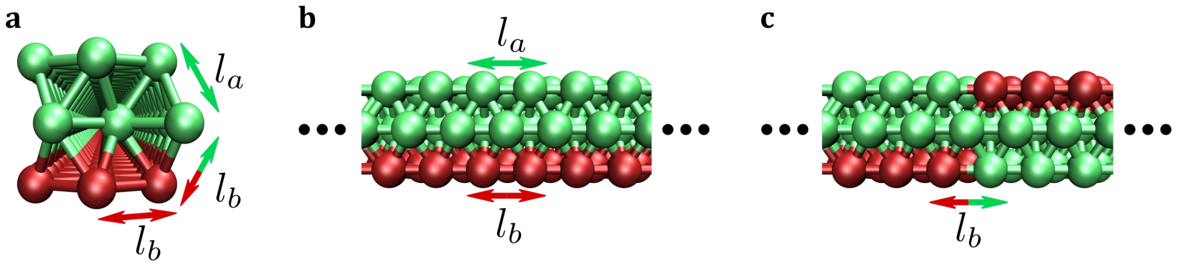

SFig. 11: **Beads arrangement used in simulations.** **a** Arrangement of beads in a section displaying in different colors the different types of bonds. **b** and **c** Front view at the position where symmetric or antisymmetric perversions occur, respectively.

To create perversions, filaments were released starting with the initial length $L = L_a = (N-1)l_a$. This means that in the initial configuration only b bonds were pre-stretched, which agrees with the experimental set-up since only one side of the filament is irradiated with UV light. During release, ends are approached at constant rate along the same direction and without allowing rotations. For sufficiently large mismatches between l_a and l_b, one or more symmetric perversions appear as discussed previously[?]. To obtain antisymmetric perversions, a and b bonds alternate positions as shown in SFig. **11c**. The same release procedure is used. Simulations were performed with time steps of $dt = 1 \times 10^{-3}$ in lj units.

In order to match the results obtained in simulations with the release experiments in **Fig. 3**, it was necessary to select three parameters as shown in Liu et al.[?]: the appropriate mismatch between l_a and l_b, the filament length L_* and the cross-section width, $w = \sqrt{3}/2(N_w - 1)l_a$. According to elastic beam theory, the longitudinal strain ϵ at an arbitrary point on the cross section of two strips is given by:

$$\epsilon = \begin{cases} \dfrac{L_* + y\theta}{L_a} - 1, y > 0 \text{ for strip } a \\ \dfrac{L_* + y\theta}{L_b} - 1, y < 0 \text{ for strip } b \end{cases} \tag{1}$$

where $L_* = \theta/K$ and θ are the length and angle of the curve, respectively. Mechanical equilibrium of forces and momenta requires:

$$\int \sigma dA = \int_0^{w_a} \sigma_a h_a dy + \int_{-w_b}^{0} \sigma_b h_b dy = 0 \tag{2}$$

$$\int \sigma y dA = \int_0^{w_a} \sigma_a y h_a dy + \int_{-w_b}^{0} \sigma_b y h_b dy = 0 \tag{3}$$

In a linear elastic material the axial stress is given by $\sigma = E\epsilon$. In computational simulations we used $h_a = 2h$ and $h_b = h$, thus equations 2 and 3 become:

$$\int_0^{w_a} 2\left(\frac{L_* + y\theta}{L_a} - 1\right) dy + \int_{-w_b}^{0} \left(\frac{L_* + \theta}{L_b} - 1\right) dy \tag{4}$$

$$\int_0^{w_a} 2\left(\frac{L_* + y\theta}{L_a} - 1\right) ydy + \int_{-w_b}^{0} \left(\frac{L_* + y\theta}{L_b} - 1\right) ydy \tag{5}$$

(6)

Neglecting the effect of the pre-strain on the width of fibres, then $w_a = w_b = w$, and hence we can write:

$$L_*\left(\frac{4}{L_a} + \frac{2}{L_b}\right) + \theta\left(\frac{2w}{L_a} - \frac{w}{L_b}\right) - 6 = 0 \tag{7}$$

$$L_*\left(\frac{6}{L_a} - \frac{3}{L_b}\right) + \theta\left(\frac{4w}{L_a} + \frac{2w}{L_b}\right) - 3 = 0 \tag{8}$$

Solving for L_* and θ, $K = \theta/L_*$ can be obtained giving:

$$K = \frac{1}{w}\frac{8\chi}{5(2+\chi)+1} \tag{9}$$

Intrinsic curvature is controlled by the pre-strain $\chi = L_a/L_b - 1$ applied to one side of the simulated fibre. SFig. **12** shows the variation of the curvature K with the pre-strain χ. Hence, for a given fibre of length L_* the number of loops can be selected by increasing or decreasing the curvature value matching experimental observations.

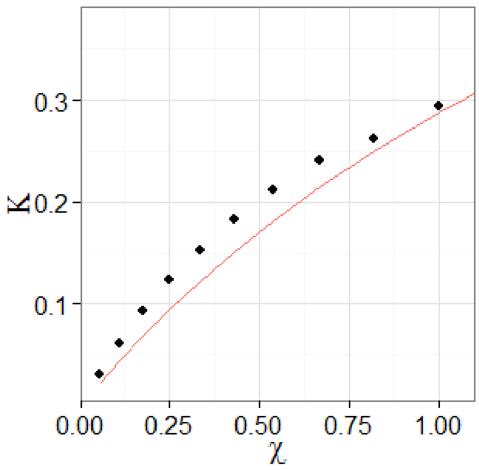

SFig. 12: **Curvature K as a function of pre-strain χ.** Elastic filaments were simulated by using pre-strained layers with $\chi = L_a/L_b - 1$. Black markers represent the curvature obtained in numerical simulations. The red line corresponds to the prediction obtained from the linear model.
